# Immunosuppressive Treatment in Children with Acquired Aplastic Anemia

**DOI:** 10.5505/tjh.2012.26779

**Published:** 2012-06-15

**Authors:** Yıldız Yıldırmak, Ela Erdem, Leyla Telhan, Laliz Kepekçi

**Affiliations:** 1 Sisli Etfal Training and Research Hospital, Department of Pediatrics, İstanbul, Turkey

**Keywords:** Immunosuppressive treatment, Acquired aplastic anemia, children

## Abstract

**Objective: **Immunosuppressive treatment (IST) is an alternative for children with acquired aplastic anemia (AA) that do not have HLA-matched donors. The objective of this study was to evaluate the outcome of IST in children with acquired AA.

**Material and Methods:** The study included 18 pediatric acquired AA patients that were retrospectively evaluated. The patients either did not have an HLA-matched related donor or were unable to undergo transplantation within 6 months despite having an HLA-matched donor.

**Results:** In all, 6 of the patients were characterized as very severe AA, 6 as severe AA, and 6 as moderate AA. Mean duration of follow-up was 44.5 months. In total, 9 patients that could not be treated with equine anti-thymocyte globulin (hATG) following diagnosis received high-dose methylprednisolone treatment. Among the 6 very severe AA patients, 2 achieved complete remission (22%); the other 16 patients received hATG+cyclosporine and short-term methylprednisolone. In total, 4 of the patients died during the first month of treatment. Of the remaining 12 patients, 3 responded to the treatment (25%). Of the 9 patients that did not respond after 3 months of treatment, 7 received a second course of immunosuppressive treatment with rabbit ATG (rATG)+cyclosporine and short-term methylprednisolone; 2 of the 7 patients responded (22%), but 5 did not respond to any treatment. Median survival among the patients was as 64 ± 8 months

**Conclusion:** Combination IST with ATG+cyclosporine and low-dose methylprednisolone was an effective treatment in the pediatric acquired AA patients with non-identical HLA donors. In the patients that couldn’t be treated with ATG high-dose methylprednisolone treatment was safe and effective.

## Abstract (tr)

**Amaç:** İmmunsupresif tedavi (İST), HLA uyumlu vericisi olmayan edinsel aplastik anemili (AA) çocuklarda diğer bir tedavi seçeneğidir. Bu çalışmada edinsel AA’li çocuklarda İST sonuçlarının değerlendirilmesi amaçlanmıştır. Gereç ve Yöntemler: Çalışmada 18 edinsel AA’li çocuk hasta retrospektif olarak incelendi. Tanı sırasında HLA uygun akraba içi vericisi olmayan ya da HLA uygun vericisi olmasına rağmen 6 ay içinde nakil olamayacak hastalar çalışmaya alındı.

**Bulgular:** Hastaların 6’sı çok ağır AA, 6’sı ağır AA ve 6’sı da orta AA sınıfında idi. Ortalama takip süresi 44.5 ay bulundu. Tanı konulduktan sonra at kaynaklı anti-timosit globulin (hATG) temin edilemeyen 9 hastaya yüksek doz metilprednizolon tedavisi verildi. Çok ağır AA’li 6 hastanın 2’sinde (%22) tam remisyon elde edildi. Geri kalan 16 hastaya hATG+siklosporin ve kısa süreli metilprednizolon tedavisi verildi.

Tedavi uygulamasını takiben bir ay içinde dört hasta kaybedildi. Geriye kalan 12 hastanın 3’ü (%25) tedaviye cevap verdi. Tedaviye başladıktan 3 ayın sonunda cevap alınamayan 9 hastanın 7’sine tavşan kaynaklı ATG (rATG)+siklosporin ve kısa süreli metilprednizolon ile ikinci kür İST verildi. Bu 7 hastanın 2’sinde (22%) cevap alındı, ancak 5 hasta tedaviye cevap vermedi. Ortalama yaşam süresi 64±8 ay bulundu.

**Sonuç:** ATG+siklosporin ve düşük doz metilprednizolon ile kombinasyon İST, HLA uygun vericisi olmayan edinsel AA’li çocuk hastalarda etkin bir tedavidir. ATG ile tedavi edilemeyen hastalarda yüksek doz metilprednizolon tedavisi güvenli ve etkilidir.

## INTRODUCTION

The primary treatment for acquired aplastic anemia (AA) is hematopoietic stem cell transplantation (HSCT) from HLA-matched sibling donors. The procedure has a survival rate of up to 88% to 97% [1]. Immunosuppressive therapy (IST) is an alternative to HSCT, especially in patients without an HLA-matched donor [1,2]. There are limited data available on the use of IST in pediatric patients; however, a large series of patients aged 1-50 years was analyzed by the European Group for Blood and Marrow Transplantation (EBMT), which concluded that HSCT should be the first-line treatment, especially in patients aged <10 years, those with severe AA, and those with an HLA-matched sibling donor [1,2]. 

Current IST options include single-dose anti-thymocyte globulin (ATG), single-dose cyclosporine (CsA), ATG+anti-lymphocyte globulin (ALG), androgens, corticosteroids, high-dose cyclophosphamide, and mycophenolate mofetil [3,4,5]. Moreover, the ATG+ALG combination is currently the most widely used regimen, although highdose cyclophosphamide therapy has been also reported [6]. The response rate to the combination of ATG+CsA is approximately 75% [7]. The aim of the present study was to evaluate the outcome of IST in pediatric acquired AA patients that could not undergo HSCT.

## MATERIALS AND METHODS

**Inclusion criteria**

Patients that presented to Sisli Etfal Training and Research Hospital, Pediatric Hematology Department between 2000 and 2008 with the diagnosis of AA were analyzed retrospectively. None of the patients had a history of using medications that can cause AA and none had hepatitis or immune-mediated disorders. All the patients included in the study had negative diepoxybutane chromosome fragility findings. Patients diagnosed as hereditary AA or those that had HLA-matched donors and underwent HSCT were excluded from the study.

**Disease classification **

AA was classified according to severity. AA was classified as severe on the basis of bone marrow cellularity <25% and the presence of ≥2 of the following criteria: neutrophil count <0.5 x 109/L, platelet count <20 x 10^9^/L, and reticulocyte count <20 x 10^9^/L. In addition to the above mentioned criteria, AA was classified as very severe if the neutrophil count was <0.2 x 10^9^/L. AA was classified as moderate in the presence of hypocellular bone marrow and any 2 of the following criteria: neutrophil count <1.0 x 10^9^/L, platelet count <50 x 10^9^/L, and reticulocyte count <60 x 10^9^/L [3,4]. 

Following diagnosis, if ATG could not be administered, high-dose methylprednisolone (HDMP) treatment was given. HDMP treatment was administered as 30 mg/kg for 3 days, and then 20 mg/kg for 4 days; after the 1st week of treatment the dose was decreased to 10 mg/kg/day for the 2nd week, 5 mg/kg/day for the 3[u]rd[/u] week, and 2 mg/kg/ day for the 4th week, after which time the treatment was withdrawn [8]. Patients received ATG if it was provided immediately after the diagnosis or if they did not respond to HDMP treatment (at least 1 month later after the beginning of HDMP treatment). The equine ATG (hATG) regimen was as follows: hATG 15 mg/kg for 5 days, CsA 5 mg/ kg/day for 60 days, and prednisolone 2 mg/kg/day for 10 days. Prednisolone dose was tapered to end on day 30. Patients that did not respond to hATG after 3 months of treatment, received ATG of rabbit origin (rATG) at a dose of 3.75 mg/kg/day for 5 days and CsA was continued at the same dosage. Prednisolone was also used at the same dosage with hATG regimen [4,9]. 

Platelets were transfused when the platelet count was <20 x 10^9^/L or when there was bleeding; erythrocyte suspension was given when the hemoglobin level was <7 g/dL and there was symptomatic anemia. 

**Response criteria **

Total response was defined as hemoglobin within agedefined normal limits, a platelet count >100 x 10^9^/L, and a neutrophil count >1.5 x 10^9^/L. Partial response was defined as a reticulocyte count >30 x 10^9^/L (independent of transfusion), a platelet count >30 x 10^9^/L, and a neutrophil count >0.5 x 10^9^/L [10]. In patients that responded to treatment, the time required for partial and complete response was analyzed. The period from diagnosis to initiation of treatment was compared between the patients that did and did not respond to the treatment. All patients underwent bone marrow aspiration at 6 and 12 months, and then yearly after the diagnosis, so that they were evaluated for myelodysplasia, which was defined as the appearance of a new clonal disorder observed cytogenetically or characteristic morphologic changes in bone marrow examination findings. 

Statistical analysis was performed using SPSS v.11.0 (Chicago, IL, U.S.A.) Descriptive statistics, including mean, median, and standard deviation, were calculated for all variables. The Mann-Whitney U test was used to compare scale variables and the survival rate was assessed via the Kaplan-Meier method.

## RESULTS

The study included 18 children that underwent IST at our hospital between 2000 and 2008. Patient demographic data are shown in Table 1. 

**Response to IST **

After the diagnosis of AA, ATG could not be given to 9 patients; instead they were given HDMP treatment. Complete response was achieved in only 2 of these 9 cases (22%), both of which had very severe AA. In total, 7 of the patients that did not respond to HDMP and 9 of patients who provided hATG received hATG, CsA, and prednisolone treatment. In all, 4 of these 16 patients died during the first month of treatment. The causes of death were infection in two patients (mucormycosis and septicemia), bleeding in one patient and ensephalopathy in the other. Of the 12 patients that survived, 3 (25%) responded to treatment, of whom 2 had moderate AA and 1 with very severe AA. In these patients who responded, CsA was stopped after tapering in 3 months. 

In total, 9 patients did not respond to 3 months of treatment; 7 subsequently received a second course of IST consisting of rATG. Prednisolone treatment was repeated and the patients received CsA treatment as the same dosage for six months. Two of the unresponsive patients chose not to receive a second course of IST; they died after 6 and 15 months of follow-up due to bleeding and infection, respectively. Among the 7 patients that underwent the second course of IST, 2 had complete response (22%) (1 with moderate AA and 1 with severe AA) and the other 5 did not respond to the second course of treatment (2 with moderate AA, 2 with severe AA, and 1 with very severe AA) (Table 2). One of the unresponsive cases died due bleeding at the end of 12-months of follow-up; the other 4 cases were still being follow-up without a response at the time this report was written. 

In the patients that responded to IST the time required for partial or complete response was analyzed. In the 2 patients that responded to HDMP the time for partial and complete response was 1.5±0.5 months and 3.5±0.5 months, respectively. Of the 3 patients that responded tohATG, partial response was obtained in 4.5±0.5 months and complete response in 12 ± 1.6 months. In the patients that received rATG as a second course following hATG,partial and complete response was achieved in 3.3±0.4 months and 11.3±0.9 months, respectively. Patientstreated with hATG became transfusion free in a meanduration of 3.7±1.4 months.Mean duration of survival in the responsive and unresponsivepatients was 76.8 ± 9.8 and months and 54.8 ±17.2 months, respectively; the difference was significant (p = 0.043). The estimated survival rate was 77% in the responsive patients and the survival rate was 51% in the unresponsive patients. Mean time from diagnosis to the start of treatment was 3.8 ± 3.8 months (range: 1 month- 12 months); among the responsive and unresponsive patients, it was 2.6±2.5 and months and 4.4 ± 4.5 months, respectively. Although the difference was significant, the patients that responded to treatment received began treatment sooner than those that were unresponsive. 

**Figure 1:**
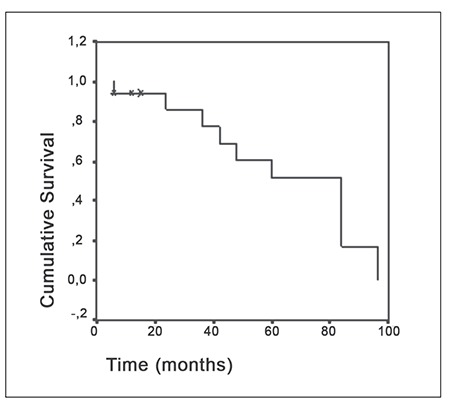
Cumulative survival rate in 18 children with aplasticanemia treated with immunosupressive therapy.

None of the patients in the present study received Granulocyte colony-stimulating factor (G-CSF). During 44.7 ± 35.2 months of follow-up none of the patients relapsed. Median duration of survival was 64 ± 8 months (range: 6-96 months) (Figure 1), and 5-year event-free survival was 61%. None of the patients exhibited evidence of clonal evolution to MDS or AML, according to bone marrow examination findings; however, cytogenetic examination of bone marrow samples is not commonly performed at the onset of disease or in unresponsive patients because of the difficulty in obtaining a sufficient number of cells for analysis.

## DISCUSSION

During the last 2 decades the use of combination IST in children with AA has increased the treatment responserate and survival time [3]. Although the peak age for thedevelopment of aplastic anemia is 15 to 25 years or olderthan 60 years, it also occurs in children [1]. Mean ageof the patients in the present study was 7.9 ± 2.5 years.Although the male:female ratio is reported to be 1:1, males predominated in the present study which was approximately 1.5:1 [1,11]. 

HDMP was given to 9 of our patients that could not be given ATG because of unavailability. Total response was achieved in only 2 of our patients. Patients with severe AA had higher survival rates with IST than other AA patients [10]. Recently, Huang et al. reported 73% overall response and 89.4% actuarial 5-year survival in children with severe AA that received IST [12]. Kurre et al. reported in a review that the combination of ATG/ALG+CsA yielded a response rate of 65% in 6 months and an overall response rate of 75% [14]. Despite the fact that the patients in the present study that responded to HDMP had very severe group AA, among those that responded to ATG and CsA, 3 had moderate AA, 1 had severe AA, and 1 had very severe AA. 

Patients that do not respond to an initial course of IST may respond to a second course of a similar or different ATG product [7]. Among the 7 patients in the present study that did not respond to the 1st course of treatment and received a 2nd course, 2 achieved total response. In all, 12 patients were treated with ATG+CsA; 5 (22%) had complete response and 7 (43%) had no response. Mean duration from the onset of treatment to remission for complete response was 12 ± 1.6 months among the patients treated with hATG and 11.3±0.9 months among those that did not respond to hATG and received rATG. 

In addition to early and late side effects caused by the use of ATG, several studies reported early deaths due to infection [9]. In the present study 2 patients had early death due to infection; 1 had severe AA and 1 had very severe AA. In patients with severe AA the early death rate due to infection is reported to be higher than in other AA patients. In the present study the early infection-related mortality rate was 11.1% (n = 2). Zheng reported that infection-related mortality was 17% during the first 6 months of IST including hATG, CsA and prednisolone [15]. Several studies reported a relapse rate of 36% following IST in AA patients, whereas when CsA was tapered slowly the rate dropped to 10%-30% [4,16]. In the present study doses were tapered slowly, which may be why relapses were not observed. Relapses following IST have occurred as late as 10 years post treatment [1]. Mean duration of follow-up in the present study was 44.7 ± 35.2 months. We think it is important to observe patients at least 10 years inorder to access the risk of relapse. 

Malignant transformation to MDS/AML is the most frequent complication of AA in patients with long-term survival; the cumulative risk is 15% in 7-8 years [7]. It remains unclear if clonal disease occurs during the natural course of AA or if it is related to IST. The patients in the present study did not develop clonal disease, which may have been due to the short-term mean follow-up of 44.7 ± 35.2 months. Long-term use of G-CSF is known to increase the risk of MDS. Although G-CSF is safe for shortterm administration, Kojima et al. reported that addition of G-CSF to IST had no benefit, in terms of hematologic response, incidence of documented infection, or overall survival in children with AA and a neutrophil count >0.2 x 10^9^L [17]. A recent review reported that although G-CSF treatment minimally increases the risk of leukemia in MDS patients and those with severe AA, a causal relationship between G-CSF treatment and risk of leukemia could not be entirely excluded [18]; therefore, we did not use G-CSF in the present study, which might explain why none of the patients developed clonal disease. 

In conclusion, the overall response rate to IST in children with AA in the present study is encouraging. The response rate obtained in the present study was lower than previously reported, which may have been because 2 patients refused a second course of IST. We think that if these 2 patients received a second course of IST, a higher overall response rate would have been obtained. Children with AA should receive IST as first-line treatment if they do not have an HLA-identical sibling donor. Combined IST with ATG, CsA, and low-dose methylprednisolone was an effective treatment option for our pediatric patients without an HLA-identical donor; however, in patients that cannot be given ATG HDMP treatment can be used as an alternative to IST, as it is an inexpensive and safe treatment option. 

## CONFLICT OF INTEREST STATEMENT

The authors of this paper have no conflicts of interest, including specific financial interests, relationships, and/ or affiliations relevant to the subject matter or materials included.

## Figures and Tables

**Table 1:**
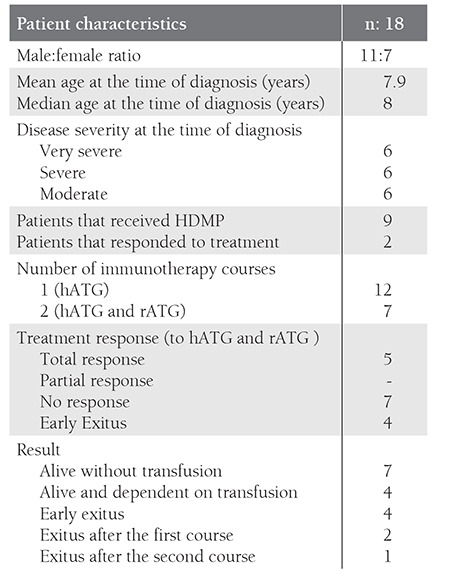
Patient demographics.

**Table 2:**
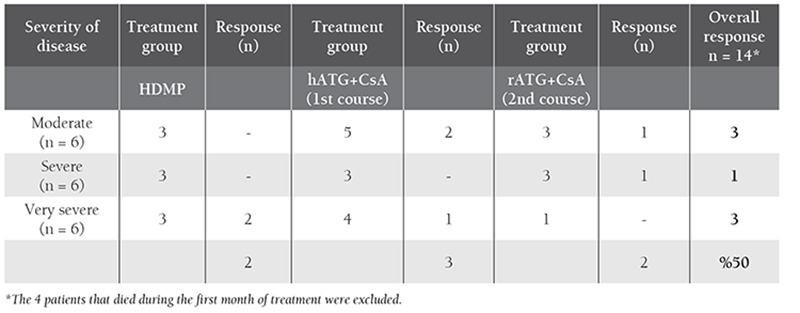
Treatment responses according to disease severity.
